# Toll-like receptors as developmental tools that regulate neurogenesis during development: an update

**DOI:** 10.3389/fnins.2014.00272

**Published:** 2014-08-28

**Authors:** Boaz Barak, Noa Feldman, Eitan Okun

**Affiliations:** ^1^McGovern Institute for Brain Research and Department of Brain and Cognitive Sciences, Massachusetts Institute of TechnologyCambridge, MA, USA; ^2^The Mina and Everard Goodman Faculty of Life Sciences, The Gonda Multidisciplinary Brain Research Center, Bar Ilan UniversityRamat-Gan, Israel

**Keywords:** TLRs (Toll-like receptors), hippocampus, neurogenesis, development, dentate gyrus, SVZ, SARM, innate immunity

## Abstract

Neurogenesis, the process of generating new neurons in the brain, fascinates researchers for its promise to affect multiple cognitive and functional processes in both health and disease. Many cellular pathways are involved in the regulation of neurogenesis, a complexity exemplified by the extensive regulation of this process during brain development. Toll-like receptors (TLRs), hallmarks of innate immunity, are increasingly implemented in various central nervous system plasticity-related processes including neurogenesis. As TLRs are involved in neurodegenerative disorders, understanding the involvement of TLRs in neurogenesis may hold keys for future therapeutic interventions. Herein, we describe the current knowledge on the involvement of TLRs in neurogenesis and neuronal plasticity and point to current knowledge gaps in the field.

## Introduction

Neuronal development is regulated by a myriad of proteins with functions intimately linked with tissue formation such as cell migration (Lakatosova and Ostatnikova, 2012), cell cycle (Borrell and Calegari, 2014) and plasticity (Park, 2013) to name a few. Surprisingly, protein families with no apparent link to neuronal plasticity are also found to be involved in neuronal development. Such is the case with the toll-like receptors (TLRs) pathway. TLRs, potent activators of the immune responses, have emerged in recent years as regulators of many aspects of neuronal plasticity and neurodegenerative processes. The aim of this mini-review is to briefly update on recent advances in our knowledge on the involvement of the TLRs pathway on developmental neuronal plasticity.

### Toll-like receptors

TLRs, hallmarks of innate immune activation (Kawai and Akira, 2007) exhibit differential expression patterns in the brain (Kaul et al., 2012) and diverse functions within the developing and adult central nervous system (CNS) (Okun et al., 2011). In this mini-review, we will review recent data that further implicates this family of innate immune receptors on CNS plasticity, with a special focus on neurogenesis. Toll was first discovered in *Drosophila melanogaster*, where it controls dorso-ventral patterning during embryonic development (Valanne et al., 2011), as well as regulates immune responses (Quintin et al., 2013). A mammalian homolog for Toll, TLR4, was later found to recognize bacterial lipopolysaccharide (LPS), a major cell wall component of Gram-negative bacteria (Okun et al., 2009). Subsequently, many additional mammalian homologs have been identified across diverse species. While *Drosophila* Toll plays both immune and developmental roles, mammalian TLRs were thought to prime an inflammatory response and facilitate activation of the adaptive immune response (Liu et al., 2010). This dogma has changed, however, and it is now recognized that mammalian TLRs also possess developmental roles during embryogenesis, as well as physiological and metabolic roles in adults. For example, TLR2 regulates multiple aspects of metabolism (Shechter et al., 2013) and is implicated, along with TLR4, in regulation of the autonomic nervous system (Okun et al., 2014). TLRs signal in both vertebrates and invertebrates in response to diverse exogenous microbial-associated molecular patterns (MAMPs) (Kawai and Akira, 2007). Endogenous TLR ligands, termed damage associated molecular patterns (DAMPs), include various extra cellular matrix components, β-defensins and heat-shock proteins (Kawai and Akira, 2007; Lehnardt et al., 2008) present during tissue damage. The signaling outcomes seem to differ between MAMP and DAMP-induced TLR activation, probably due to the need to balance between immune intervention and tissue damage repair (Liu et al., 2009).

### Neurogenesis

**Figure 1:**
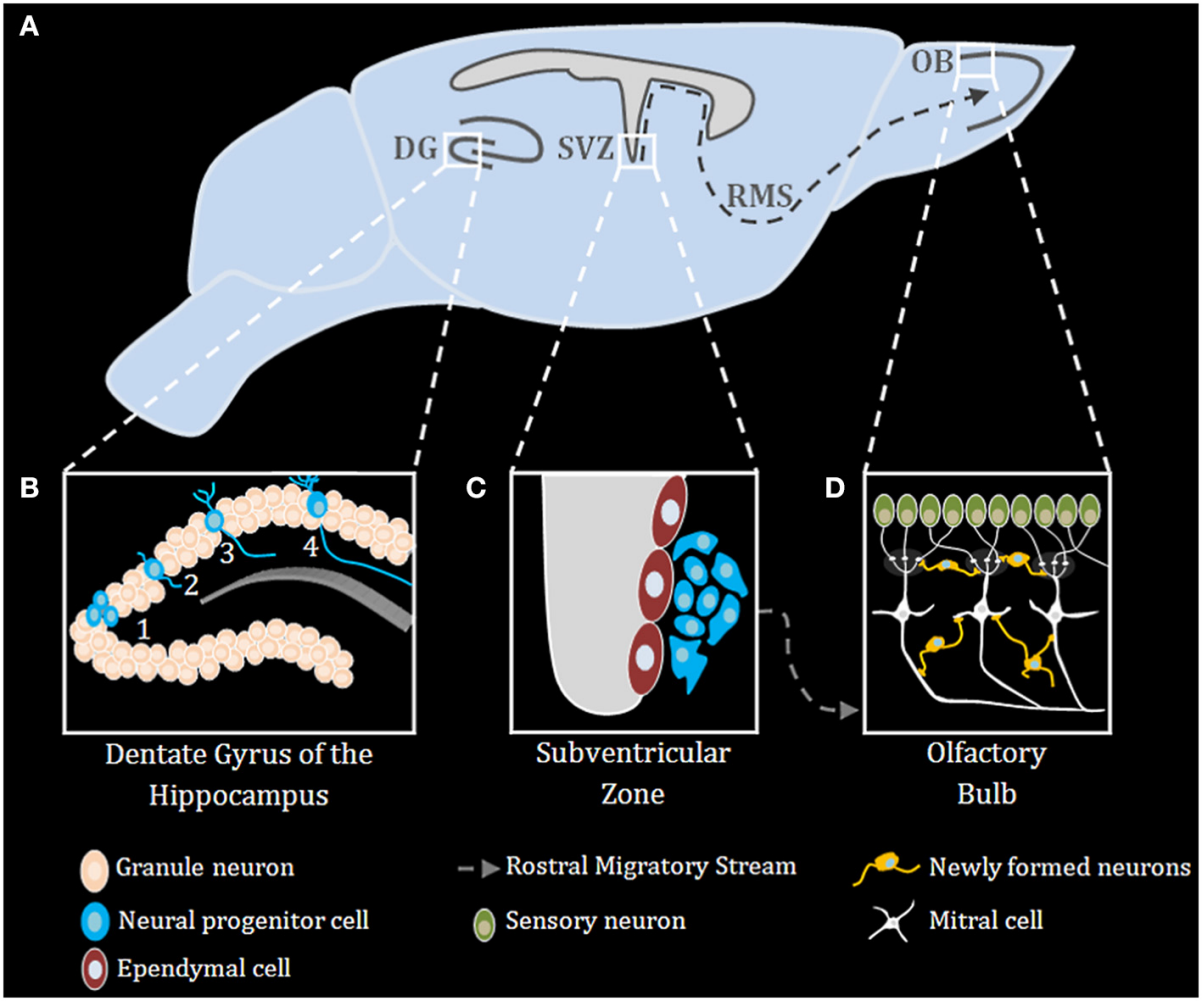
**Adult neurogenesis in the mouse brain**. **(A)** Midsagittal view of the mouse brain with the three main regions of adult neurogenesis: dentate gyrus (DG) of the hippocampus, subventricular zone (SVZ) and the olfactory bulb (OB) **(B)** Adult neurogenesis in the DG of the hippocampus is generally divided into four stages: (1) proliferation, (2) migration, (3) differentiation, (4) integration. **(C)** Stem cells proliferating in the SVZ migrate via the rostral migratory stream (RMS) to the OB **(D)**, where they differentiate and finally integrate into neural circuits.

Neurogenesis occurs robustly during embryogenesis, and diminishes during early post-natal development and into adulthood (Duan et al., 2008). Improper neurogenesis results in severe embryonic developmental brain abnormalities (e.g.: microcephaly) (Barkovich et al., 2012; Kahoud et al., 2014). Successful neurogenesis requires proper execution of multiple steps: neuronal progenitor cells (NPC) proliferation, migration, differentiation, maturation, and ultimately functional integration of newly formed neurons into existing neuronal networks. During mammalian embryonic development, extensive neurogenesis occurs in the sub ventricular zone (SVZ) of the lateral ventricles (Sakamoto et al., 2014) (Figure 1). In adults, neurogenesis in rodents is restricted to the subgranular zone (SGZ) of the hippocampal dentate gyrus (DG) and the sub ependymal zone (SEZ) lining the lateral ventricles (Figure 1), however, in humans it was recently shown that neurogenesis also occurs in the striatum (Ernst et al., 2014). NPCs in the nervous system can self-renew and differentiate into all types of neural cells, including neurons, astrocytes, and oligodendrocytes (Gage, 2000) (Figure 1). Neurons arising from the SGZ, differentiate and integrate locally into the DG as granule cells, which ultimately act as primary excitatory neurons in the DG, contributing to both formation and extinction of new memories (Akers et al., 2014). New neurons formed in the SEZ, however, migrate through the rostral migratory stream (RMS) to the olfactory bulb where they contribute to plasticity in the olfactory system (Sakamoto et al., 2014) (Figure 1). Numerous endogenous signaling pathways (for an extensive review see Sasai and Yamamoto, 2013) regulate the neurogenic niche signals. As described below, TLRs emerge as an important family of such endogenous factors regulating CNS plasticity in general and neurogenesis in particular.

### TLR expression dynamics during brain development

**Figure 2:**
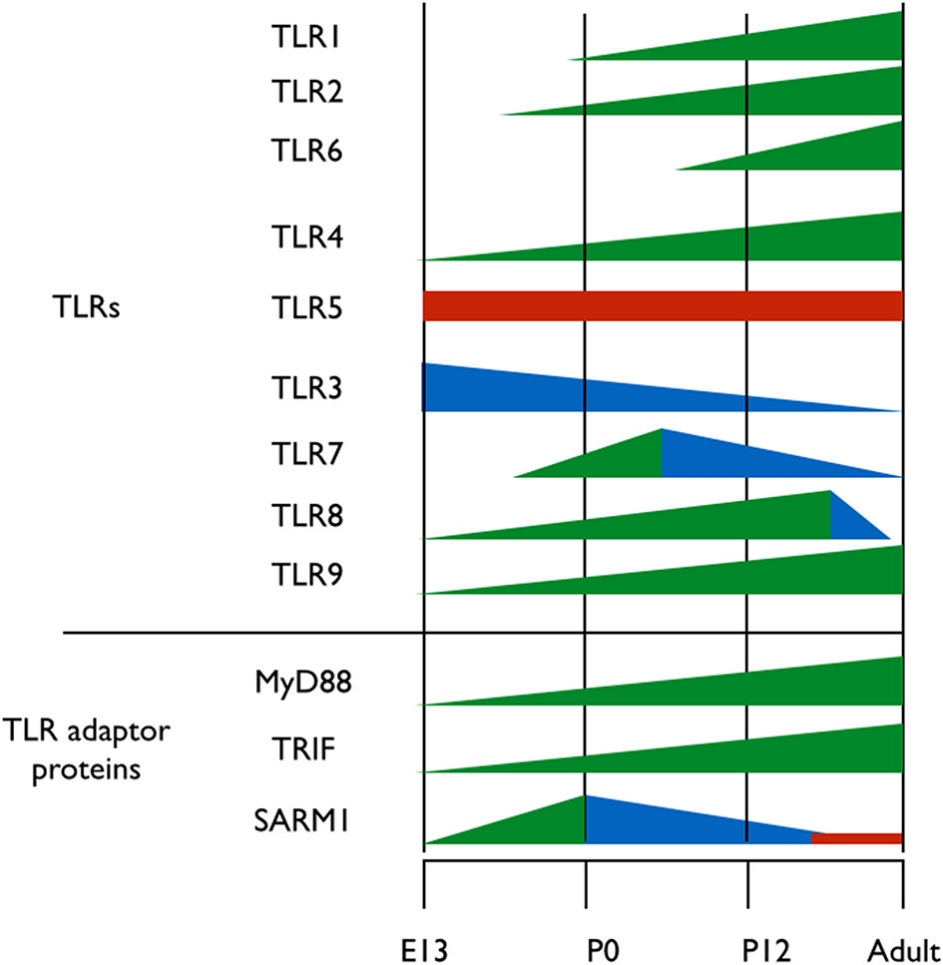
**Expression pattern of TLRs and their adaptor proteins throughout brain development**. TLRs 1-9 and the TIR adaptor proteins MyD88, TRIF, and SARM1 were thus far analyzed for their protein expression levels during brain development. Different colors represent changes in proteins expression levels; green represents increased expression, blue represents decreased expression and red represents stable expression.

A clue to understanding the roles of TLRs in neuronal plasticity and neurogenesis can be obtained by analyzing the expression pattern of TLR-related genes during brain development. Kaul et al. (2012) have recently performed the first comprehensive attempt at assessing the mRNA expression levels of TLR-related genes throughout development. However, spatial information on the expression of those genes in the developing brain is still lacking. It is clear, however, that the expression of the various TLR protein family members is differentially regulated throughout development; TLR2 and its heterodimer partners, TLR1 and TLR6 are expressed at early postnatal days (Okun et al., 2010b; Kaul et al., 2012) (Figure 2). TLR4, which functions as a homodimer, gradually increases in expression from early embryonic stages and maintains high expression level in the brain during adulthood (Lathia et al., 2008; Kaul et al., 2012) (Figure 2). TLR5 forms asymmetric homodimers (Zhou et al., 2012) and seems to maintain a stable expression level throughout the developmental process (Kaul et al., 2012) (Figure 2). Nucleic acid sensing TLRs also exhibit differential expression pattern during development; TLR3 expression is already in its highest levels in the early period of cortical development when NPC are highly proliferative (Lathia et al., 2008) (Figure 2). Its expression then declines as neurogenesis and gliogenesis ensues and low expression levels are maintained in the adult (Kaul et al., 2012) (Figure 2). TLR7 expression levels in the developing mouse transiently increase at the time of birth, and gradually declines afterwards as the animal develops (Kaul et al., 2012) (Figure 2). TLR8 expression in the brain can be detected as early as embryonic day 12, following which it increases until dramatically declines after postnatal day 21, which is when the basic patterns of neurogenesis and axonogenesis are complete. Thus, expression levels of TLR8 in the adult brain are low (Ma et al., 2006a,b; Kaul et al., 2012) (Figure 2). Finally, TLR9 expression constantly increases during late embryogenesis and postnatal stages until adult levels are reached and remains stable (Kaul et al., 2012) (Figure 2).

TLRs signal via 4 Toll/IL-1 receptor (TIR) adaptor proteins, namely, MyD88, Mal, Trif, TRAM and one inhibitory TIR adaptor protein, SARM (O'Neill and Bowie, 2007). The importance of understanding the expression pattern of these adaptor proteins extends beyond the scope of TLRs, as MyD88, for example, also mediates signaling from the interleukin (IL)-1 receptors super-family (Janssens and Beyaert, 2002). Of the five known TIR adaptor proteins, only MyD88, TRIF, and SARM were systemically analyzed for expression during brain development, and the expression pattern of Mal and TRAM remains to be determined. Analysis of TRIF mRNA levels reveals a gradual increase in expression until birth, after which TRIF mRNA levels decrease again (Kaul et al., 2012) (Figure 2). The expression levels of MyD88 during embryogenesis are unclear, as one study found that MyD88 expression remains relatively constant following birth (Kaul et al., 2012), while a second study found that MyD88 levels decrease (Okun et al., 2010b) (Figure 2). During mouse embryonic development, SARM protein expression levels increases gradually in the brain, peaking on embryonic day 18, a period of significant neuronal proliferation and programmed cell death occurs, followed by a threefold decrease in expression levels after birth (Kim et al., 2007). The fact that distinct TLRs and their TIR adaptor signaling proteins exhibit specific and distinct expression patterns during brain development suggests a physiological relevance of specific TLRs to brain development.

### The TLR pathway in neural development

With the realization that TLRs are expressed in the CNS (Ma et al., 2006b; Rolls et al., 2007; Tang et al., 2007), a significant effort was put into understanding the functions of TLRs in brain-residing cells. As summarized below, the TLR pathway exhibits pleiotropic effects on neuronal plasticity including neurogenesis during brain development. The relevancy of TLR-related genes expression to developmental neuronal plasticity is discussed, rather than microbial-mediated TLR activation, which has been extensively reviewed elsewhere (Okun et al., 2009, 2011).

#### NPC proliferation

Deficiency for TLR2 in both adult and embryonic stages does not affect the proliferative capacity of NPC (Rolls et al., 2007; Okun et al., 2010b). Deficiency for TLR1 and TLR6, binding partners for TLR2, was not yet tested, and therefore it remains an open question whether these TLR members affect neurogenesis. TLR3-deficiency enhances the proliferative capacity of embryonic but not adult NPC, correlated with diminished TLR3 expression during development (Lathia et al., 2008). Deficiency for TLR4 increases NPC self-renewal (Rolls et al., 2007). SARM1 was shown to inhibit TRIF-dependent TLR3 and TLR4 signaling in immune cells (Carty et al., 2006; O'Neill and Bowie, 2007). While developmental deficiencies for either TLR3 or TLR4 were shown to increase embryonic NPC proliferation in the SVZ, it is unlikely that these effects are related to SARM1, as SARM1 is not expressed in NPC in the embryonic SVZ (Lin et al., 2014b). There is no information on the impact of TLR5, 7, 8 or 9 on NPC proliferation, and this remains an open question.

#### NPC differentiation

The effects of TLR2 on NPC differentiation are developmental stage-dependent. The differentiation of embryonic NPC is not affected by TLR2 deficiency (Okun et al., 2010b). Within the adult brain, TLR2 is expressed in both the SEZ and SGZ neurogenic niches, and specifically on cells that co-express the early neuronal marker, doublecortin (DCX), glial fibrillary acid protein (GFAP), myeloid cells or NPC (Rolls et al., 2007; Okun et al., 2010b). In contrast to the embryonic brain, at this stage, TLR2 does affect the fate of adult hippocampal NPC differentiation. Specifically, TLR2 deficiency promotes astrocytic rather than neuronal fate in differentiating NPC (Rolls et al., 2007). NPC from TLR4-deficient mice or NPC treated with siRNA against TLR4, exhibit a higher proportion of neurons at the expense of astrocytes (Rolls et al., 2007). These cells, however, do not survive to become mature neurons *in vivo*, implying that additional survival signals must act in concert with TLR4 to successfully execute the neurogenesis process (Rolls et al., 2007). A similar effect is observed in MyD88-deficient mice, suggesting that MyD88 mediates the effects conferred by TLR4 on neurogenesis (Rolls et al., 2007). Consistent with an inhibitory role for TLR3 on NPC proliferation, neurogenesis is increased in TLR3-deficient mice, correlating with increased DG volume (Okun et al., 2010a). Overall, our understanding of the effects of TLRs on NPC differentiation is limited to studies conducted on TLRs 2, 3, and 4. The field is open for additional studies that will enhance our insights on the roles of innate immune receptors in general and TLRs in particular in neurogenesis.

#### Axonal growth

**Figure 3:**
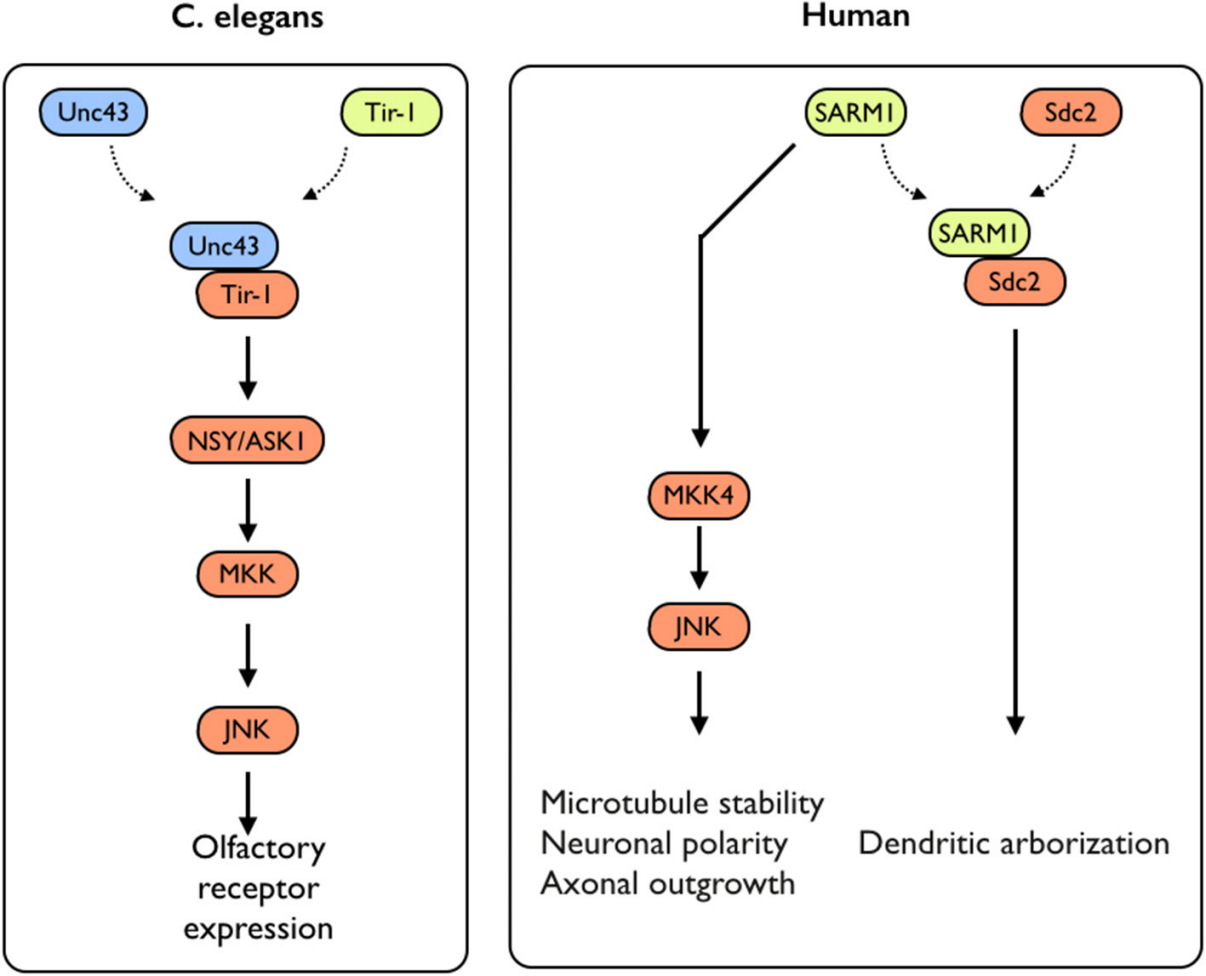
**SARM1-mediated signaling in *C. elegans* and humans**. In *C. elegans*, Sarm1/Tir-1 has been shown to regulate left–right asymmetric expression of odorant receptor genes in olfactory neurons via association with Unc43 (homolog to the mammalian CamKII), which leads to downstream activation of the NSY/MKK/JNK signaling pathway. In mammals, Sarm1 was shown to play multiple roles in controlling neuronal morphology. Sarm1 physically interacts with transmembrane heparan sulfate proteoglycan syndecan-2 (Sdc2) and mediates the function of Sdc2 to control dendritic arborization. In addition, Sarm1 also regulates microtubule stability, establishment of neuronal polarity and axonal outgrowth. These functions are likely mediated through the MKK4/JNK pathway.

While several studies have addressed the impact of TLR-activation on axonal growth during neuronal differentiation, not much is known on the impact of the expression of TLR-related genes on this process. An exception to that is the sterile α and TIR motif–containing protein 1 (Sarm1), a TLR-related adaptor protein, which was recently studied for its roles in neuronal plasticity. Sarm1 is a multidomain adaptor molecule containing two sterile α motifs and one Toll/interleukin-1 receptor homology domain. Sarm1 was originally identified in humans as a negative regulator of the TRIF-dependent TLR3 and TLR4 pathways in innate immunity (Mink et al., 2001; Carty et al., 2006). Importantly, Sarm1 is known to function in the nervous system; Toll and interleukin 1 receptor domain protein (Tir-1), an ortholog of Sarm1 in *Caenorhabditis elegans*, is highly concentrated at synapses (Chuang and Bargmann, 2005). Tir-1 receives synaptic signals via interaction with CaMK, regulating the downstream ASK1–MKK–JNK pathway, further regulating olfactory receptor expression (Figure 3) (Chuang and Bargmann, 2005). Unlike the other TIR domain-containing adaptor proteins, Sarm1 is predominantly expressed in the mammalian brain (Lin et al., 2014b) and preferentially expressed in neurons, where it also regulates neuronal survival by targeting JNK3 to the mitochondria (Kim et al., 2007). In the brain, Sarm1 interacts with and receives signals from syndecan-2 (Sdc2), a synaptic heparan sulfate proteoglycan that triggers dendritic filopodia and spine formation as well as regulates dendritic arborization in cultured hippocampal neurons through the MKK4–JNK pathway (Figure 3). Sarm1-deficient mice exhibit reduced dendritic arborization compared to wild-type littermates (Chen et al., 2011). In addition to acting downstream of Sdc2, Sarm1 is required for proper initiation and elongation of dendrites, axonal outgrowth, and neuronal polarization (Chen et al., 2011). These functions likely involve Sarm1-mediated regulation of microtubule stability, as Sarm1 influences tubulin acetylation (Chen et al., 2011). Moreover, Sarm1-deficient mice also exhibit a higher spine density on hippocampal CA1 dendrites, in a mechanism involving mGluR5 (Lin et al., 2014a). Perhaps of the highest importance with respect to axonal growth, both *Drosophila* SARM1 (dSarm) and murine SARM1 suppress Wallerian degeneration cell-autonomously for weeks after axotomy. This indicates that the functionality of Sarm1 as a pro-degenerative agent is conserved in mammals (Osterloh et al., 2012). Several TLRs with significant impacts on neuronal plasticity, including neurogenesis and axonal growth, were shown to exhibit pleiotropic effects on mouse cognitive behavior (Okun et al., 2010a, 2012) (for an extensive review see Okun et al., 2011). Similarly, SARM1 deficiency exhibits multiple effects on various aspects of cognitive behavior (Lin and Hsueh, 2014). This includes a mild impairment effect on spatial learning (tested using a T-maze) and impaired contextual-fear learning. Interestingly, social interaction is also impaired in these mice (Lin and Hsueh, 2014). It is not clear yet whether these effects are solely developmental or whether Sarm1 affects adult cognitive behavior independently of its effect on neuronal plasticity. Moreover, it is not known whether the pleiotropic effects mediated by SARM1 are part of a larger TLR3- or TLR4-dependent endogenous activation or whether SARM1 mediates these effects in a TLR-independent manner.

## Conclusions

TLRs (mostly TLRs 2, 3, and 4) and their adaptor proteins (MyD88, Sarm1) exhibit distinct effects on the different aspects of neurogenesis throughout the development of the brain. This may not come as a surprise, given the critical role of the *Drosophila* Toll gene during the embryonic development of the fly, and the immunological function it later undertakes in the adult fly (Valanne et al., 2011). As indicated in the “open question” section, it will be interesting to uncover whether other members of the TLR family are involved in neurogenesis and what mechanism underlies these effects downstream to TLRs in the brain.

### Open questions in the field:

- TLR2 typically forms heterodimers with TLR1 or TLR6. While a deficiency of TLR2 has no impact on NPC proliferation, it is not known whether deficiency of TLR1 or TLR6 affects NPC proliferation.
- The impact of TLRs 5, 7, 8 or 9 has not yet been examined for an effect of NPC proliferation.
- So far, the only TIR adaptor protein examined for their expression pattern during brain development were Trif, MyD88 and Sarm1. It is interesting to examine the expression pattern of Mal and TRAM during brain development. Further, it will be interesting to assess whether these adaptor proteins have an active role in brain development.
- Developmental deficiency for Sarm1 exhibits pleiotropic effects on cognitive behavior. It is not clear, however, whether these effects are solely developmental or whether Sarm1 affects adult cognitive behavior independently of its effects on neuronal plasticity.

### Conflict of interest statement

The authors declare that the research was conducted in the absence of any commercial or financial relationships that could be construed as a potential conflict of interest.
